# Supplementary material for: Multicentric study of cervical cancer screening with human papillomavirus testing and assessment of triage methods in Latin America: the ESTAMPA screening study protocol
Source: BMJ Open. 2020 May 24;10(5):e035796. doi: 10.1136/bmjopen-2019-035796 (PMC7252979; doi:10.1136/bmjopen-2019-035796)
Supplement: Supplementary data [file bmjopen-2019-035796supp001.pdf]

Sample size calculation
ESTAMPA study

Sample size was computed for testing differences in proportions of sensitivity for HSIL+ of paired-sample triage tests according to equation (3) presented by Connor RJ (1987).[1]

We combined several differences of the proportion of HSIL+ cases positive for a pairwise of triage tests (sensitivities for HSIL+) at several levels of pairwise mismatch. In a 2 × 2 table of HSIL+ paired-samples (see below), we established a proportion of discordance between a pair of triage tests in one direction, λ_{10}, and then using a given difference of sensitivity (d) we established the proportion of discordance in the opposite direction as $\lambda_{01} = |\lambda_{10} - d|$

		**Triage test A**	
		Positive=1	Negative=0
**Triage test B**	Positive=1	λ_{11}	λ_{10}
	Negative=0	λ_{01}	λ_{00}

Then, we computed the sample size using equation (3) from Connor RJ.[1]

For example: given a pairwise discordance of $\lambda_{10} = 0.09$ (λ_{10} direction) and difference of sensitivity of $d = 0.05$, the discordance in the opposite direction would be $\lambda_{01} = 0.04$ and the number of HSIL+ would be $n = 406$ if 80% power or $n = 542$ if 90% power, for 0.05 type I error.

The following figure represents the sample size with 0.05 type I error, and 80% and 90% power in function of d at different levels of pairwise discordances (λ_{10}):

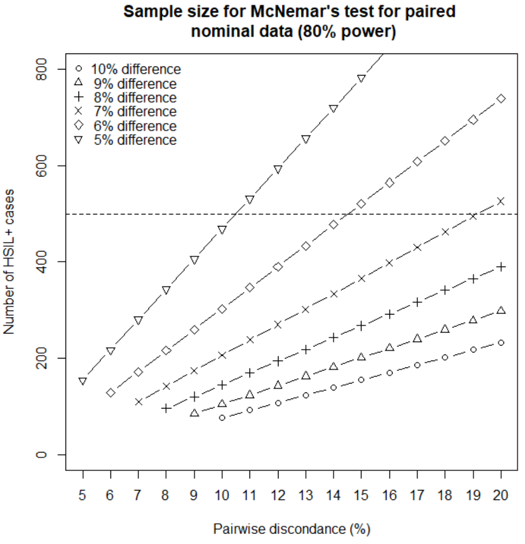

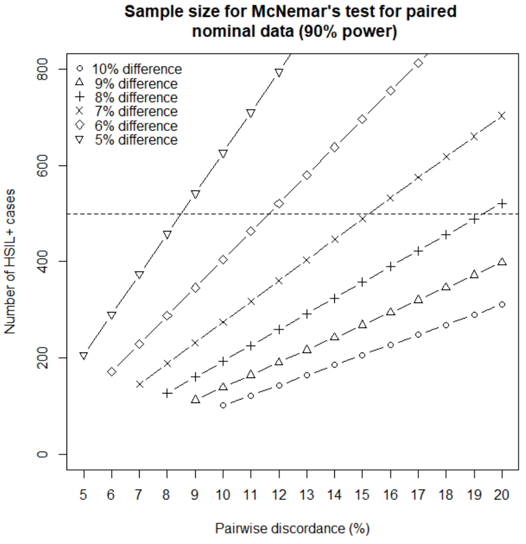

The ESTAMPA study will be able to identify at least 500 HSIL+ cases based on 1% prevalence. The following figure represents the power for a sample size of 500 HSIL+ cases and 0.05 type I error in function of d at different levels of pairwise discordances (λ_{10}).

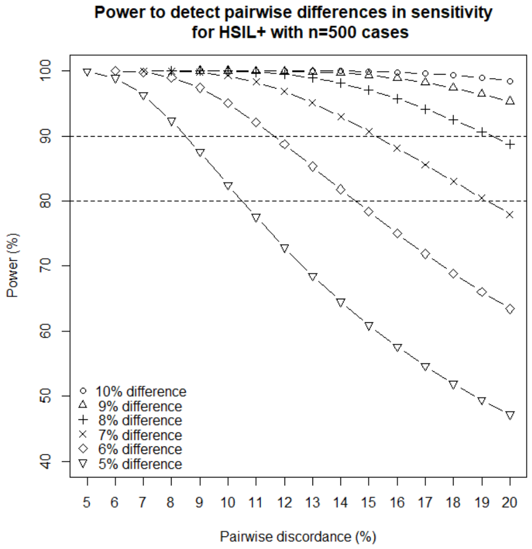

Therefore, with 0.05 type I error, we will be able to detect a 5% difference in sensitivities for the detection of HSIL+ between two pared triage tests for pairwise discordances up to 10% with 80% power or up to 8% pairwise discordance with 90% power.

We are also planning to compare some triage tests or several combinations between them. Therefore, the type I error and the statistical power to detect a certain difference in sensitivities between several paired triage tests at a certain pairwise discordance would be affected. The greater the number of tests compared, the lower the power. The following figure presents the power to detect 5% differences in sensitivities affected by multiple comparisons using Bonferroni correction.

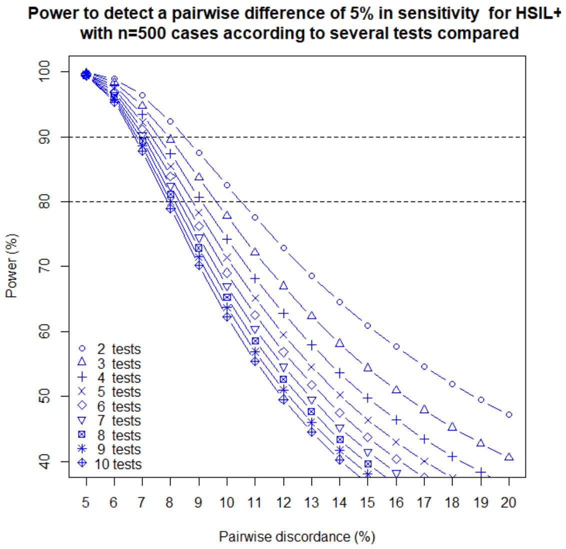

For up to 10 comparisons, the power will remain approximately above 80% only if the pairwise discordance is lower than 8%.

Inter-centre heterogeneity could also affect the statistical power and comparisons between the triage tests. However, posteriori adjustments will be done during the analysis phase to include this feature in the results.

Reference

1. Connor RJ. Sample size for testing differences in proportions for the paired-sample design. *Biometrics* 1987;43(1):207-11.
