# Supplementary material for: Multicentric study of cervical cancer screening with human papillomavirus testing and assessment of triage methods in Latin America: the ESTAMPA screening study protocol
Source: BMJ Open. 2020 May 24;10(5):e035796. doi: 10.1136/bmjopen-2019-035796 (PMC7252979; doi:10.1136/bmjopen-2019-035796)
Supplement: Supplementary data [file bmjopen-2019-035796supp002.pdf]

List of all institutions obtaining ethical approval for the ESTAMPA study

INSTITUTION	LATEST DATE OF APPROVAL
International Agency for Research on Cancer (IARC) Ethics Committee (IEC), Lyon, France	29/03/19 *
Pan American Health Organization Ethics Review Committe (PAHOERC), Washington DC, USA	22/05/13
Instituto Nacional de Cancerología (INC) Ethics Committe, Bogotá, Colombia	31/03/15
Universidad de Antioquia, (U de A), Medellín Bioethics Committee, Colombia	24/02/16
Instituto de Investigaciones en Ciencias de la Salud (IICS) Ethics Committee, Universidad Nacional de Asunción (UNA), Asunción, Paraguay	15/09/14
Universidad nacional autónoma de Honduras (UNAH), Tegucigalpa, Honduras	29/10/14
Instituto Nacional de Donación y Transporte de Células, Tejidos y Organos, Bioethics Committee, Minsitry of Health, Uruguay	05/11/15
Caja Costarricense del Seguro Social, CCSS, Ethics Committee, San Jose, Costa Rica	11/08/14
Instituto Nacional de Salud Pública de México (INSP), Ethics Committee, Morelos, México	26/05/16
Ministerio de Salud Pública y Bienestar Social de la República del Paraguay (MSPBS), Ethics Committee, Asunción, Paraguay	06/03/15
Hospital Nacional Profesor Alejandro Posadas, Ethics Committee Buenos Aires, Argentina	12/04/18
Hospital Clínicas José de San Martín, Buenos Aires, Argentina	28/11/14
Universidad San Martin de Porres, Facultad de Medicina Humana, Ethics Committee, Lima, Perú	28/05/18
Servicio Departamental de Salud Chuquisaca, Ethics Committee, Sucre, Bolivia	09/05/16

* The first version of the protocol was approved by IEC on 25/09/13, after the protocol was approved by PAHOERC on 22/05/2013.
